# Intravascular Ultrasound Observation of the Mechanism of No-Reflow Phenomenon in Acute Myocardial Infarction

**DOI:** 10.1371/journal.pone.0119223

**Published:** 2015-06-02

**Authors:** Junxia Li, Longmei Wu, Xinli Tian, Jian Zhang, Yujie Shi

**Affiliations:** Department of Cardiology, Military General Hospital of Beijing People’s Liberation Army Hospital, Beijing, 100700, China; Northwestern University Feinberg School of Medicine, UNITED STATES

## Abstract

**Objective:**

To study the mechanism of the no-reflow phenomenon using coronary angiography (CAG) and intravascular ultrasound (IVUS).

**Methods:**

A total of 120 patients with acute myocardial infarction (AMI) who successfully underwent indwelling intracoronary stent placement by percutaneous coronary intervention (PCI). All patients underwent pre- and post-PCI CAG and pre-IVUS. No-reflow was defined as post-PCI thrombolysis in myocardial infarction (TIMI) grade 0, 1, or 2 flow in the absence of mechanical obstruction. Normal reflow was defined as TIMI grade 3 flow. The pre-operation reference vascular area, minimal luminal cross-sectional area, plaque cross-sectional area, lesion length, plaque volume and plaque traits were measured by IVUS.

**Results:**

The no-reflow group was observed in 14 cases (11.6%) and normal blood-flow group in 106 cases (89.4%) based on CAG results. There was no statistically significant difference in the patients’ medical history, reference vascular area (no-flow vs. normal-flow; 15.5 ± 3.2 vs. 16.2 ± 3.3, p> 0.05) and lesion length (21.9 ± 5.1 vs. 19.5 ± 4.8, p> 0.05) between the two groups. No-reflow patients had a longer symptom onset to reperfusion time compared to normal blood-flow group [(6.6 ± 3.1) h *vs* (4.3 ± 2.7) h; p< 0.05] and higher incidence of TIMI flow grade< 3 (71.4% vs 49.0%, p< 0.05). By IVUS examination, the no-reflow group had a significantly increased coronary plaque area and plaque volume compared to normal blood-flow group [(13.7 ± 3.0) mm^2^ vs (10.2 ± 2.9) mm^2^; (285.4 ± 99.8) mm^3^ vs (189.7 ± 86.4) mm^3^; p< 0.01]. The presence of IVUS-detected soft plaque (57.1% vs. 24.0%, p< 0.01), eccentric plaque (64.2% vs. 33.7%, p< 0.05), plaque rupture (50.0% vs. 21.2%, p< 0.01), and thrombosis (42.8% vs. 15.3%) were significantly more common in no-reflow group.

**Conclusion:**

There was no obvious relationship between the coronary risk factors and no-reflow phenomenon. The symptom onset to reperfusion time, TIMI flow grade before stent deployment, plaque area, soft plaques, eccentric plaques, plaque rupture and thrombosis may be risk factors for the no-reflow phenomenon after PCI.

## Instruction

The purpose of the acute myocardial infarction (AMI) treatment strategy is to rescue the dying heart by completely opening the occluded blood vessels. In recent years, studies have shown that more than 25% of myocardial tissue blood flow does not completely recover with revascularization, even in some patients who achieve thrombolysis in myocardial infarction (TIMI) flow grade of 2 or lower at least 10 min after the end of the PCI procedure in coronary angiography (CAG) imaging [1–3]. This lack of blood flow recovery is known as the no-reflow phenomenon. The no-reflow phenomenon weakens the clinical benefit of emergency percutaneous coronary intervention (PCI). Compared with normal blood flow patients, these patients often have cardiac insufficiency, post-infarction angina and other complications [4, 5]. Therefore, studying the mechanism of the no-reflow phenomenon is important. Currently, coronary angiography can reflects changes in the vascular lumen to predict the risk of no-reflow. Several studies have demonstrated that biomarkers of coronary lumen changes could also have a prognostic role in the prediction of the no-flow phenomenon [1–5]. Intravascular ultrasound (IVUS) is a recently developed interventional technique that not only reflects changes in the vascular lumen, it reflects the cross-sectional structure of the blood vessels, including plaques and plaque traits [6, 7]. IVUS plays an important role in the evaluating characteristic lesion morphology and plaque composition with the no-reflow phenomenon [8, 9]. The aim of the present study is to use IVUS to explore the mechanism of the no-reflow phenomenon by measurements of coronary artery structure changes, plaque morphology, and plaque composition.

## Materials and Methods

### Ethics statement

This study was approved by the institutional review board of the Military General Hospital of Beijing People’s Liberation Army Hospital. All participants provided their written consent to participate in this study.

### Patient population

From January 2008 to December 2012, we identified a total of 120 patients with a first AMI who underwent IVUS interrogation before PCI and were eligible for enrollment. All patients were treated by stent deployment within 12h after the onset of symptoms. Patients with medical histories of cerebral hemorrhage, massive cerebral infarction within 3 months, active visceral bleeding within 1 month, and intolerance with dual anti-platelet therapy (aspirin and clopidogrel) were excluded for this study. IVUS catheter failed to pass through the targeted lesions/plaques were excluded for this study too.

### PCI procedure

CAG and primary PCI were performed according to the standard guidelines with antiplatelet therapy and administration of periprocedural anticoagulants [1–3]. All patients were prescribed aspirin (loading dose, 200 mg) plus clopidogrel (loading dose, 300 or 600 mg) before or during PCI. All infarct lesions were treated with stent implantation (sirolimus-eluting stents, Cypher stent, Cordis, Johnson and Johnson, Miami Lakes, FL).

### Pre- and post-PCI CAG examinations

A Philips H3000 digital cardiac imaging system (Philips Medical System Nederland; Veenpluis 4–6, 5680 DA Best) was used at an acquisition speed of 15 frames/s for angiography. All patients underwent PCI through the upper extremity arteries or femoral artery. Culprit vessels (infarct-related arteries) were defined as those vessels corresponding to elevated surface ECG ST-segments, acute occluded blood vessels or vessels with visual thrombosis in the ultrasound images [9]. A computer-aided cardiovascular angiography analysis system was used to analyze the angiography data and to perform TIMI flow grade statistics immediately after coronary stenting [10]. No-reflow was defined as a residual stenosis <10% after responsible coronary stent implantation with no vascular dissection, while maintaining a forward blood flow ≤TIMI flow grade 2 [11].

### Pre-PCI IVUS examination

A 3F 30-MHz IVUS catheter CLEARVIEW (Boston Scientific, Boston, Massachusetts) was advanced to the distal culprit artery, and the insertion method was similar to PCI surgery. Specifically, when CAG was accomplished, ordinary heparin sodium (70 u/kg) was injected into the artery, a 0.014-in guide-wire was imported to the distal culprit artery, and the IVUS catheter was guided along with the guide-wire to the distal end of the examined coronary artery. Full 360-degree cross-sectional images of blood vessels were obtained by an automatic back-to-the-point system at a track speed of 1 mm/s. Patients with either spontaneous revascularization or no revascularization were required to complete each IVUS examination. If the IVUS catheter failed to pass through the lesion, the patient was excluded from the study. IVUS images were initially saved in a medical DICOM format and then recorded onto CDs or other digital media for offline analysis. Two experienced physicians who were unaware the clinical data and CAG images of the patients assessed the IVUS images separately. Computer software analysis was used to quantitatively and qualitatively diagnose coronary artery disease with parameters like the reference segment vessel area, total vessel cross-sectional area (VA), minimal luminal cross-sectional area (MLA), plaque cross-sectional area (PA, PA = VA-MLA), and lesion length. With playback IVUS images, we automatically generated the vascular volume (VV) and lumen volume (LV) using software after continuous measurement of the vascular area and lesion length measurements. The plaque volume was also calculated (PV; PV = VV-LV). Plaque traits included calcified plaque, hard plaque, and soft plaque, the degree of plaque eccentricity, fibrous cap thickness, and the presence or absence of a lipid pool. The ultrasound echo from a calcified plaque is stronger than the echo from the vascular adventitia, which is accompanied by an acoustic shadow and the sonic range > 90°. The echo from a hard plaque is similar to the vascular adventitia echo, but no acoustic shadow is present. The echo of a soft plaque appears weaker than the vascular adventitia echo. Thrombosis showed homogeneity with loose flocculence spots and scintillation echoes based on the low echo. A plaque rupture lacuna attached to the lumen lacuna results in a visible residual fibrous cap [12, 13].

### Statistical Analysis

Statistical analyses of the measured data were performed using SPSS10.0 software; all numerical data were expressed as the mean ± SEM (the standard error of the mean). The averages from the two groups were compared by t tests. Enumeration data were compared by *x* ^2^ tests and p< 0.05 was considered statistically significant.

## Results

### Patient characteristics and Quantitative CAG analysis

Using Quantitative CAG analysis, no-reflow was defined as post-PCI TIMI grade 0, 1, or 2 flow in the absence of mechanical obstruction. Normal reflow was defined as TIMI grade 3 flow [14, 15]. On this basis, patients were divided into two groups, a no-reflow group (n = 14, 11.6%) and a normal reflow group (n = 106, 88.4%95). The differences in the medical history (no-flow vs. normal-flow; hypertension 35.7% vs.36.5%; diabetes 42.8% vs. 37.7%; hyperlipidemia 57.1% vs. 58.6%; smoking 35.7% vs. 37.5%; pre-infarction angina 57.1% vs. 59.6%; and creatine phosphate muscle enzyme peak value 2255 ± 1210 vs. 2143 ± 1322) in the two groups were not statistically significant (all p> 0.05) (Table 1).

**Table 1:** Comparison of clinical data and coronary lesions between the 2 groups.

	No-reflow (n = 14)	Normal Blood flow (n = 106)	*x* ^*2*^ value	*p* value
Age	64.6 ± 10.1	62.7 ± 10.2	0.66	> 0.05
Female	6 (42.8%)	43 (40.5%)	0.03	> 0.05
Diabetes	6 (42.8%)	40 (37.7%)	0.14	> 0.05
Hypertension	5 (35.7%)	38 (36.5%)	0.00	> 0.05
Smoking	5 (35.7%)	39 (37.5%)	0.00	> 0.05
Hyperlipidemia	8 (57.1%)	61 (58.6%)	0.00	> 0.05
Pre-infarction angina	8 (57.1%)	62 (59.6%)	0.00	> 0.05
Enzyme peak (u/L)	2255 ± 1210	2143 ± 1322	0.32	> 0.05
Symptom onset to reperfusion time (h)	6.6 ± 3.1	4.3 ± 2.7	2.64	< 0.05
TIMI0 Grade before stenting	10 (71.4%)	51 49.0%)	2.68	< 0.05

The symptom onset to reperfusion time was longer in the no-reflow group, and the difference between the two groups was statistically significant (p< 0.05) and a higher incidence of TIMI flow grade 0 before treatment was found in the no-reflow group, and the difference between the two groups was statistically significant (p< 0.05) (Table 1).

### IVUS analysis

The reference vascular area (no-flow vs. normal-flow; 15.5 ± 3.2 vs. 16.2 ± 3.3, p> 0.05) and lesion length (21.9 ± 5.1 vs. 19.5 ± 4.8, p> 0.05) between the two groups. No-reflow patients had a longer symptom onset to reperfusion time compared to normal blood-flow group [(6.6 ± 3.1)h *vs* (4.3 ± 2.7)h; p< 0.05] and higher incidence of TIMI flow grade< 3 (71.4% vs 49.0%, p< 0.05). The no-reflow group had a significantly increased coronary plaque area and plaque volume compared to normal blood-flow group [(13.7 ± 3.0) mm^2^ vs (10.2 ± 2.9) mm^2^; (285.4 ± 99.8) mm^3^ vs (189.7 ± 86.4) mm^3^; p< 0.01] (Table 2). The presence of IVUS-detected soft plaque (57.1% vs. 24.0%, p< 0.01), eccentric plaque (64.2% vs. 33.7%, p< 0.05), plaque rupture (50.0% vs. 21.2%, p< 0.01), and thrombosis (42.8% vs. 15.3%) were significantly more common in no-reflow group. The difference of calcified plaque between the two groups was not statistically significant (p> 0.05) (Table 3).

**Table 2:** Comparison of coronary lesions measured by IVUS between the 2 groups.

	No-reflow (n = 14)	Normal Blood flow (n = 106)	t value	*p* value
Reference vascular area (mm^2^)	15.5 ± 3.2	16.2 ± 3.3	0.76	> 0.05
Lesion length (mm)	21.9 ± 5.1	19.5 ± 4.8	1.66	> 0.05
Plaque area (mm2)	13.7 ± 3.0	10.2 ± 2.9	4.11	< 0.01
Plaque volume (mm3)	285.4 ± 99.8	189.7 ± 86.4	3.42	< 0.01

**Table 3:** Comparison of coronary lesions traits in IVUS between the 2 groups.

	No-reflow (n = 14)	Normal Blood flow (n = 106)	*x* ^*2*^ value	*p* value
Calcified plaque	6 (42.8%)	40 (38.5%)	0.14	> 0.05
Soft plaque	8 (57.1%)	25 (24.0%)	6.98	< 0.01
Eccentric plaque	9 (64.2%)	35 (33.7%)	5.20	< 0.05
Plaque rupture	7 (50.0%)	22 (21.2%)	5.71	< 0.05
Thrombosis	6 (42.8%)	16 (15.3%)	6.36	< 0.05

**Fig 1:**
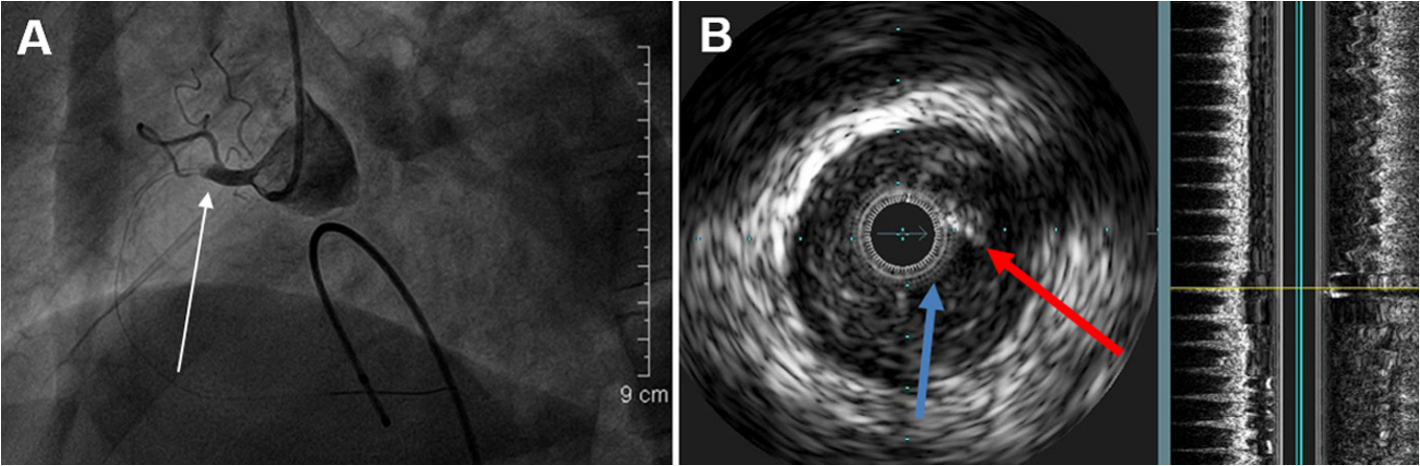
Pre-CAG and IVUS of a reprehensive no-reflow case. A 55-year-old male patient was admitted for "acute inferior myocardial infarction". Emergency CAG revealed the occlusions of the proximal right coronary artery (arrow, Fig 1A). IVUS detected the thrombosis shadow (red arrow) and thin fibrous cap (blue arrow); the longitudinal image served as the localization of the plaque (yellow line) (Fig 1B).

**Fig 2:**
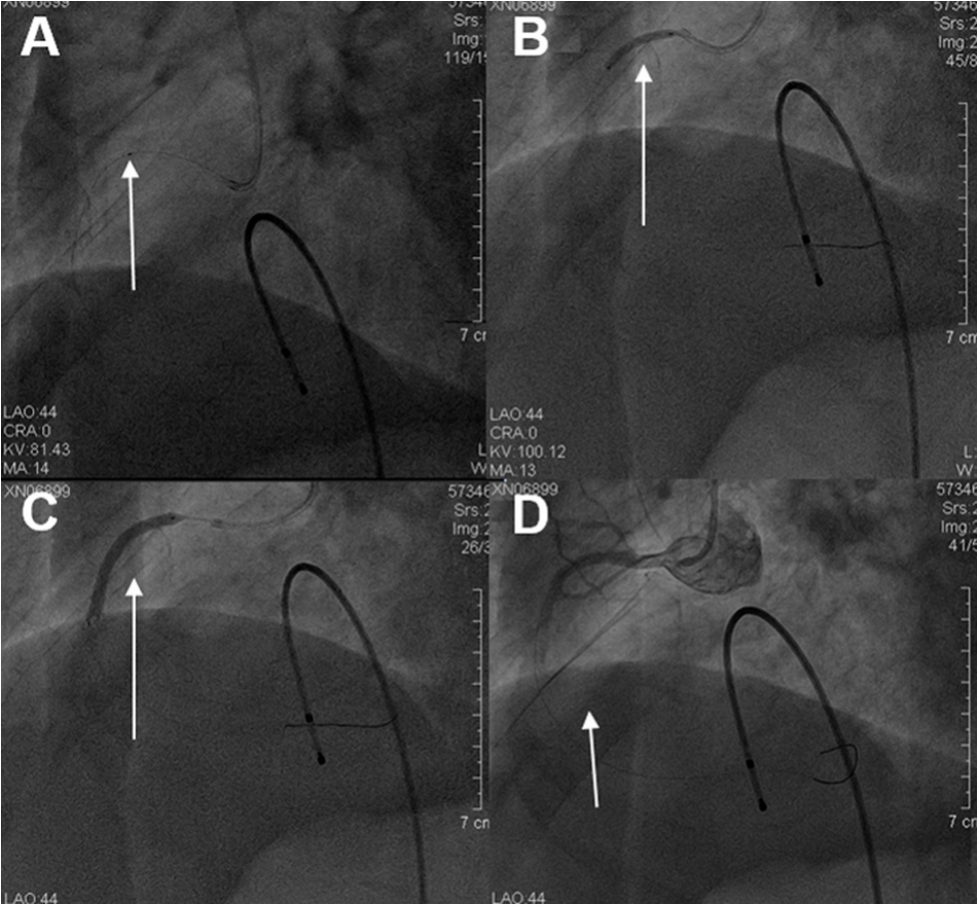
PCI intervention therapy of the same patient in Fig 1. Coronary thrombus aspiration was shown (arrow, Fig 2A), balloon dilatation (arrow, Fig 2B), stent implantation (arrow, Fig 2C), and no-reflow imaging (arrow, Fig 2D).

Fig 1 showed a reprehensive no-reflow case. A 55-year-old male patient with "acute inferior myocardial infarction". Emergency CAG revealed occlusions of the proximal right coronary artery (Fig 1A). IVUS detected the thrombosis shadow, thin fibrous cap and longitudinal image of the plaque (Fig 1B). Fig 2 showed PCI procedure of the same patient in Fig 1, the patient underwent coronary thrombus aspiration (Fig 2A), balloon dilatation (Fig 2B), stent implantation (Fig 2C), and no-reflow imaging (Fig 2D).

## Discussion

IVUS findings can explain the mechanism of the no-reflow phenomenon by monitor coronary artery morphology and the characteristics of plaque composition. CAG is considered the gold standard for diagnosing coronary artery disease. However, CAG can only estimate the coronary lumen size [16]. IVUS not only reflects changes in the vascular lumen, it also reflects the cross-sectional structure of the blood vessels, including plaques [17]. Plaque detection, IVUS and pathological diagnosis are very well correlated. IVUS can be used to produce real-time, low-magnification pathologic images of vascular structures because IVUS has a high sensitivity and specificity for detecting plaque ruptures and thrombosis [17]. IVUS is an important tool for investigating the mechanism of coronary heart disease and determining the interventional treatment [18].

The aim of reperfusion treatment of PCI for AMI is to quickly and fully restore myocardial blood flow to avoid further myocardial necrosis and dysfunction; therefore, no-reflow after PCI affects the clinical treatment efficacy [14, 15]. No-reflow is generally defined as a residual stenosis< 10% after responsible coronary stent implantation with no vascular dissection and little to no advanced blood perfusion into the myocardium [19]. As a complication, no-reflow can lead to blood pressure decreases and cardiogenic shock as well as increased in-hospital mortality and re-MI [20]. No-reflow is also a predictor of continued ischemia, infarction extension, ventricular remodeling and cardiac dysfunction as well as a mark of serious cardiac and microvascular injury [21]. Currently, angiography is the most common and invaluable method for detecting no-reflow [16]. However, using forward flow ≤ TIMI flow grade 2 as a categorization method for evaluating coronary, no-reflow does not fully explain its mechanism [22]. It is possible that coronary a peripheral thrombus or the plaque composition causes occlusion, myocardial microcirculation disorders, cardiac syncope, and ischemia-reperfusion injury (e.g., freedom injury, vascular endothelial tissue injury, inflammatory cell infiltration, and myocardial edema) [23, 24]. With the development of coronary and myocardial perfusion and coronary morphology evaluation tools, an increasing number of studies show that micro-thrombi (15 ~ 100μm) caused by unstable plaque ruptures in the intervention process play a very important role in the mechanism of myocardial reperfusion [25, 26].

Clinical observations show that the age, heart failure, symptom onset to reperfusion time, collateral circulation, pre-infarction angina, Q-wave count, wall motion score and ST-segment elevations are related to coronary heart disease events and the clinical prognosis of the patient after emergency PCI [27]. These factors also affect the functional status of ischemic myocardial tissue microcirculation, but the exact relationship to the no-reflow phenomenon is still unclear.

In this study, the incidence of no-reflow phenomenon was 11.6%, and the no-reflow phenomenon occurred in patients who had larger thrombi or plaques. The symptom onset to reperfusion time was longer in the no-reflow group than that in normal blood-flow group (p< 0.05). The primary reason for this was that the time to hospital in the no-reflow group was later. A higher incidence of TIMI flow grade 0 before treatment was found in the no-reflow group than that in normal blood-flow group (p< 0.05). The reference vascular area and lesion length between the two groups were no signicant differences. No-reflow patients had a significantly longer symptom onset to reperfusion time compared to normal blood-flow group and a higher incidence of TIMI flow grade< 3 was found. The no-reflow group had a significantly increased coronary plaque area and plaque volume compared to normal blood-flow group. The soft plaque, eccentric plaque, plaque rupture, and thrombosis were significantly more common in no-reflow group compared to normal blood-flow group. The difference of calcified plaque between the two groups was not statistically significant. This indicates that the mechanism of no-reflow may be involved in the interventional treatment, which leads to thrombotic or plaque components into peripheral blood vessels and causes thrombosis. A previous study showed that a porridge-like substance from the coronary artery could be drawn using the Rescue thrombus aspiration catheter in no-reflow patients with acute coronary syndrome after successful PCI, and the pathology analysis showed that the aspiration tissue contained many platelets, high fibrinogen levels and many cholesterol crystals [28]. This observation demonstrates that the no-reflow phenomenon is not only related to thrombosis, it is related to the composition of atherosclerotic plaques, which appear as hypoechoic plaques in IVUS. Tanaka et al conducted IVUS in 100 patients with AMI [1]. The findings from this study suggested that plaques appear as eccentricities, lipid pool-like images, plaque ruptures, dissections, positive vascular remodeling and other features. Patients with slow blood flow had the aforementioned characteristics, and 13 no-reflow phenomenon cases showed large lipid pool shadows in the blood vessels and plaque burden with microcirculation disturbance after PCI. Nakamura et al.’s study reported consistent results and verified this observation [29]. There are other reports that the no-reflow phenomenon of IRA after PCI is correlated with positive vascular remodeling and plaque volume reduction, indicating that a component of the no-reflow phenomenon may be related to distal vascular embolization [30, 31]^.^ Therefore, preoperative IVUS and coronary angiography are important for preventing peripheral vascular thrombosis in patients with larger thrombus lesions or plaque areas. In addition, it is very important to consider adequate anticoagulation and the use of devices that prevent distal embolization. Although some attempts have been made to reduce slow flow after PCI, such as with the use of distal protection devices that reduce distal microvascular thrombosis, the current randomized clinical trials have not shown positive results [32]. Drug prevention in clinical practice may have some value. A study by Shinozaki et al directly performed PCI in patients with AMI and used sodium nitroprusside to reduce the occurrence of slow flow or no-reflow before balloon dilatation [33]. Because sodium nitroprusside can improve microcirculation, slow coronary flow phenomena may be related to microcirculation disturbances.

No-reflow is also related to the reperfusion time, suggesting the important concept of "time is life, time is myocardial viability" [34]. It is crucial in clinical practice to perform emergency PCI green channels and making efforts to shorten the ischemic time. It is generally accepted that the larger the infarct area, the higher the incidence of no-reflow. Our study observed there is on definite relationship between the infarct area and no-reflow by myocardial enzyme peak observation. The use of ischemic preconditioning mechanisms may improve microcirculation and reduce the no-reflow phenomenon in pre-infarction angina [23]. However, the relationship between ischemic preconditioning mechanisms and the no-reflow phenomenon requires further studies to confirm our results.

AMI therapy not only requires rapid and sustained vessel opening but also requires recovering the microvascular blood flow and myocardial tissue perfusion, which necessitates more effective reperfusion methods and strategies.
